# Psychological Distress, Personality Traits and Functional Disability in Patients With Osteonecrosis of the Femoral Head

**DOI:** 10.14740/jocmr1851w

**Published:** 2014-07-28

**Authors:** Odysseas D Mouzas, Aristidis H Zibis, Konstantinos S Bonotis, Crysanthos D Katsimagklis, George M Hadjigeorgiou, Maria N Papaliaga, Apostolos P Dimitroulias, Konstantinos N Malizos

**Affiliations:** a Department of Psychiatry, Faculty of Medicine, School of Health Science, University of Thessaly, Greece; b Department of Orthopaedics, Faculty of Medicine, School of Health Science, University of Thessaly, Greece; c Department of Neurology, Faculty of Medicine, School of Health Science, University of Thessaly, Greece

**Keywords:** Osteonecrosis, Psychological distress, Functional disability, Personality traits

## Abstract

**Background:**

The aim of the present study was to investigate personality traits, psychological distress and functional disability in patients with non-traumatic osteonecrosis of the femoral head (ONFH).

**Methods:**

Sixty-seven patients participated in the study, 48 males and 19 females. The mean age was 37.6 years (SD: 10.92, range: 15 - 61). Seventy-five healthy individuals, age and sex matched, served as controls. Socio-demographic information and clinical data were collected. The following instruments were used: the General Health Questionnaire (GHQ-28), the Defence Style Questionnaire (DSQ) and the World Health Organization Disability Assessment Schedule II (WHO-DAS II).

**Results:**

Patients suffering from ONFH presented higher scores at the GHQ-28 compared to healthy controls (P < 0.001). Duration of disease (P < 0.047) and age (P < 0.023) were the main factors associated with psychological distress (P < 0.003). Personality traits such as image distorting (P < 0.025) and self-sacrificing (P < 0.029) were identified in patients with ONFH compared to healthy controls. Functional disability was associated with high scores at GHQ-28 scale (P < 0.001). The “adaptive personality structure”, as measured by DSQ was negatively associated with functional impairment (P < 0.022).

**Conclusions:**

Patients with ONFH more commonly present symptoms of psychological distress associated with distinct functional clinical parameters. The present study also reveals the role of personality traits. Further investigation could specify the possible influence of psychopathology and personality traits or coping strategies on the course of disease.

## Introduction

Osteonecrosis of the femoral head (ONFH) is a disabling clinical entity with approximately 20,000 new cases reported each year in the United States [1, 2]. It commonly affects young individuals in their late 30s and early 40s, a productive age range with increased needs of mobility and functionality [3, 4]. The natural history of ONFH, in the great majority of cases, proceeds through distinct stages, ultimately resulting in collapse of the subchondral bone and destruction of the articular surface of the hip joint [2, 5, 6]. This subsequently leads to pain and limping with significant restriction of the range of hip motion, and joint destruction. Without specific treatment, 80% of clinically diagnosed cases will progress and will eventually require total hip arthroplasty [2]. ONFH is the underlining diagnosis in as many as 10% of 500,000 total hip replacements performed every year in the United States [7].

While the research for the etiology of ONFH reveals new causative factors, ONFH most often is characterized as secondary and is associated with numerous different pathological conditions [8]. ONFH may be established in the course of, or after the treatment of a number of diseases. Consequently, patients with ONFH may often have the experience of more than one disease. In the majority of the cases both femoral heads are involved and in about 60% of patients cerebral white matter lesions (WMLs) have been observed with magnetic resonance imaging. This condition is designated as white matter lesions in osteonecrosis (WMLeON) [9]. Besides, necrotic lesions may also affect other joints such as the knee, the shoulder (humeral head) and the ankle (talus), further aggravating the physical disability of the patient. For these reasons, ONFH has been characterized as a chronic systemic disease rather than a hip disorder [9].

Although evidence suggests that depression contributes to the disability associated with chronic illnesses [10], to our knowledge, there are no research studies investigating the extent to which various clinical, demographic, or personality features could be associated with possible psychological distress in ONFH patients. Similarly, there are no studies focusing either on the clinical parameters associated with functional disability, or on a range of psychopathological conditions, such as symptomatic anxiety and depression, in patients with ONFH.

The present study aims at identifying the possible association of the clinical parameters of ONFH with certain personality traits and aspects of psychological distress, as well as the functional disability of these patients. For these purposes, a wide range of clinical, psychological and demographic parameters were investigated. Screening and dimensional instruments for the assessment of functional disability, as well as for the detection of various psychological distress symptoms were administered. Additionally, defence styles were assessed, in order to identify the structural personality traits of patients with ONFH and define their relationship to psychiatric morbidity.

## Materials and Methods

Sixty-seven patients diagnosed with ONFH were prospectively followed at the Orthopaedic Department of University Hospital of Larissa in Greece.

The participants were 48 males and 19 females with a mean age of 37.6 years (SD: 10.92, range: 15 - 61). The mean duration of illness was, at the time of evaluation, 20.0 months (SD: 10.5, range: 2 - 43). In 71.7% of patients, both hips were involved and in 48.8%, at least one osteonecrotic lesion in the skeleton was present, additional to those of the femoral heads. In 14.5% of cases, the ONFH was idiopathic and in 85.5%, it was secondary. Regarding the secondary ONFH, all patients underwent detailed medical history evaluation about the primary disease. The severity of the primary disease was classified, according to the American Society of Anaesthesiologists (ASA) criteria. Thirty patients with osteonecrosis (44.8%) were classified as ASA stage I or II and 37 (55.2%) as stage III or IV.

Since the goal of the present study was to identify factors affecting psychological distress in patients with ONFH, it was necessary to distinguish the factors that might be associated with psychological distress in the general population from those possibly associated with distress among patients with ONFH. For this reason, 75 individuals randomly selected from the hospital’s staff list and medical students served as the healthy control group. They were not manifesting problems requiring medical or psychiatric intervention, were free of any medication at the time of investigation and did not have a history of psychotic illness, alcohol and/or drug abuse, or dementia.

### Instruments and procedures

For the assessment of parameters such as psychological distress, personality traits and physical disability, the following instruments were used.

#### General Health Questionnaire (GHQ-28)

The GHQ-28 was used for the assessment of psychopathology [11]. GHQ-28 is a self-administered screening questionnaire, designed to detect probable psychopathology in primary care settings. It can estimate the likelihood of having a psychiatric diagnosis or disease to those administered. It is a reliable and valid questionnaire widely used in clinical research [11, 12]. It examines four factors (somatic symptoms of depression, anxiety and insomnia, social dysfunction and depressive feelings). For the purposes of this study, the translated and adapted for the Greek population version [13] was used. According to the standardization of GHQ-28 for the Greek population, the best cut-off score point of the Greek version is 5. As the total GHQ-28 score exceeds this recommended cut-off score point, the probability of being assessed as having a psychiatric diagnosis at interview increases. The traditional GHQ method of scoring “0011”, devised by Goldberg, was carried out. Subsequently, the alternative Likert scoring method “0123” was carried out for the four clusters of psychopathology. GHQ-28 has been widely used in patients with somatic diseases (autoimmune, unknown etiology like systemic sclerosis (SS), etc.) and studies have shown that it may be used as a valid instrument for screening as well as for assessing the impact of illness on these kinds of diseases [14].

#### Defence Style Questionnaire (DSQ)

The DSQ is a rating scale that is designed to estimate behavior suggestive of 25 ego defence mechanisms, which are psychodynamic in origin, and four defence styles, namely “maladaptive action”, “image distorting”, “self-sacrificing” and “adaptive” [15]. Maladaptive action style consists of apparent derivatives of withdrawal, regression, acting out, inhibition, passive aggression and projection defence mechanisms and indicates the participants’ inability to deal with their impulses by taking constructive action on their own behalf. Image distorting style consists of apparent derivatives of omnipotence, splitting and primitive idealization defences, and the essence of this style is the splitting of the image of self and other into good and bad and into strong and weak. Self-sacrificing style consists of apparent derivatives of reaction formation and pseudoaltruism defence mechanisms and reflects a need to perceive one’s self as being kind, helpful to others and never angry. Finally, adaptive style consists of apparent derivatives of suppression, sublimation and humor and is associated with good coping [15]. DSQ was translated into Greek, and standardized for Greek population [16].

#### Greek version of the World Health Organization Disability Assessment Schedule II (WHO-DAS II)

The Greek version of the WHO-DAS II was used to assess the functional profile of the patients. The WHO-DAS II is a validated, multidimensional questionnaire, which is conceptually compatible with the framework of the International Committee of Functioning, Disability and Health [17]. It was designed to assess the activity limitations and participation restrictions actually experienced by an individual, irrespective of diagnosis. The 36-item WHODAS II contains 32 questions covering six domains of assessment: understanding and communicating, getting around, self-care, getting along with people, life activities and participation in society [18]. Using multi-item scales, it assesses a variety of impairment and disability dimensions like: work loss days, pain, concentration, mobility, self-care, family burden, social participation and discrimination [19]. The final scores range from 0 to 100 with higher scores indicating greater disability. Row scores were translated to a scale from 0 to 100, using an SPSS syntax (available through the WHO).

### Statistical analysis

Mean values ± standard deviation were used to describe quantitative variables, while absolute and relative frequencies were used to describe qualitative variables. Log-transformation was made for GHQ depression scale because of its skewed distribution. Chi-square test was used for the comparison of proportions. For the comparison of means between osteonecrosis patients and control group, Student’s *t*-tests were used. Pearson correlations coefficients were used to explore the association of two continuous variables. Multiple linear regression analysis was conducted in order to find independent factors associated with GHQ and DSQ scales in patients with osteonecrosis. Age, sex, duration of disease, the presence of multiple lesions, ASA classification and scores on WHO-DAS II were entered as independent variables in the models. Regression coefficients and standard errors were computed from the results of the linear regression analysis. Diagnostics for regression models were performed to check if the conditions for regression had been met with the residuals of each model being normally distributed and their variance being constant. All reported P values are two-tailed. Statistical significance was set at P < 0.05 and analysis was conducted using SPSS statistical software (version 17.0).

## Results

The sample consisted of 67 patients with osteonecrosis and 75 controls. The control group was similar in terms of age (35.5 ± 12.7 vs. 37.6 ± 10.9, P = 0.288) and sex (men: 68% vs. 71.6%, P = 0.637) with the osteonecrosis group. Characteristics of the osteonecrosis group are presented in Table 1.

**Table 1:** Characteristics of the Osteonecrosis Group

	N	%
Sex		
Men	48	71.6
Women	19	28.4
Age (years), mean ± SD	37.6 ± 10.9	
Duration of disease, mean ± SD	20.0 ± 10.5	
Multiple lesions		
No	42	62.7
Yes	25	37.3
ASA		
I-II	30	44.8
III-IV	37	55.2
WHODASII, mean ± SD	27.3 ± 21.0	

Twenty-five patients (37.3%) had multiple lesions. Table 2 shows the mean values of GHQ and DSQ scales for the osteonecrosis and control group. Greater values on “somatic symptoms of depression”, “social dysfunction” and “total score” were found in the osteonecrosis group compared with the control group. As for the DSQ scales, mean values of “image distorting” and “self-sacrificing” scale were greater for the osteonecrosis group.

**Table 2:** Comparison of GHQ and DSQ Scales Between the Osteonecrosis and the Control Group

	Osteonecrosis group (N = 67)	Control group (N = 75)	
	
	Mean ± SD	Mean ± SD	P Student’s *t*-test
GHQ			
Somatic symptoms	11.7 ± 3.7	9.5 ± 2.6	< 0.001
Anxiety	12.7 ± 4.3	12.1 ± 3	0.321
Social dysfunction	14.9 ± 4	8.8 ± 2.5	< 0.001
Feelings of depression	9.2 ± 3.1	10.1 ± 2.8	0.040
Total score	48.4 ± 11.8	40.5 ± 9	< 0.001
DSQ			
Maladaptive action	126.1 ± 41	120.2 ± 18.5	0.265
Image distorting	58.8 ± 21.8	52.2 ± 12.1	0.025
Self-sacrificing	39.8 ± 10.4	36 ± 10.3	0.029
Adaptive	39.3 ± 9.4	36.6 ± 7.9	0.064

Correlation analysis (Table 3) between GHQ and DSQ scales in patients with osteonecrosis showed a significant positive association between “maladaptive action” and “anxiety scale”. Also, “maladaptive action” and “image distorting” were positively associated with “feelings of depression”. All intercorrelations between GHQ scales or DSQ scales were significant.

**Table 3:** Correlations Coefficients Between GHQ and DSQ Scales in Patients With Osteonecrosis

	Somatic symptoms	Anxiety	Social dysfunction	Feelings of depression	GHQ total score	Maladaptive action	Image distorting	Self-sacrificing
Anxiety	0.55***							
Social dysfunction	0.50***	0.33**						
Feelings of depression	0.43***	0.60***	0.49***					
GHQ total score	0.80***	0.80***	0.75***	0.78***				
Maladaptive action	0.02	0.25*	-0.03	0.28*	0.12			
Image distorting	-0.09	0.16	-0.18	0.32*	0.02	0.70***		
Self-sacrificing	0.02	0.16	-0.11	0.11	0.05	0.46***	0.50***	
Adaptive	-0.09	-0.08	-0.15	-0.01	-0.11	0.43***	0.40***	0.34**

*P < 0.05; **P < 0.01; ***P < 0.001.

When multiple linear regression analysis was conducted with dependent the GHQ scales (Table 4), it was found that WHO-DAS II was independently and positively associated with all dimensions of GHQ. Furthermore, duration of disease was positively associated with “somatic symptoms of depression”, “feelings of depression” and “total score” of GHQ. Multiple analysis revealed that older age was accompanied with greater levels of anxiety and GHQ total score. Additionally, greater levels of anxiety were found in patients with multiple lesions, while greater levels of feelings of depression were found in patients with III or IV ASA classification, compared to those with I or II ASA classification.

**Table 4:** Multiple Linear Regression Models for GHQ Scales: Regression Coefficients ± Standard Error

	Somatic symptoms		Anxiety		Social dysfunction		Feelings of depression		Total score	
	β ± SE*	P	β ± SE	P	β ± SE	P	β ± SE	P	β ± SE	P
Sex										
Men, reference										
Women	0.21 ± 1.13	0.852	1.29 ± 1.27	0.316	-1.67 ± 1.17	0.163	-0.04 ± 0.04	0.347	-1.15 ± 3.09	0.711
Age (years)	0.02 ± 0.05	0.687	0.16 ± 0.06	0.008	0.03 ± 0.05	0.526	0.00 ± 0.01	0.693	0.30 ± 0.13	0.023
Duration of disease	0.03 ± 0.02	0.046	0.04 ± 0.05	0.424	0.03 ± 0.05	0.504	0.01 ± 0.001	0.043	0.09 ± 0.05	0.047
Multiple lesions	0.46 ± 0.93	0.625	2.0 ± 1.0	0.048	1.13 ± 0.96	0.248	0.01 ± 0.03	0.662	1.15 ± 2.54	0.652
ASA										
I-II, reference										
III-IV	0.35 ± 0.98	0.722	0.96 ± 1.10	0.388	0.67 ± 1.02	0.517	0.03 ± 0.01	0.049	1.38 ± 2.68	0.609
WHODASII	0.10 ± 0.02	< 0.001	0.08 ± 0.03	0.008	0.15 ± 0.03	< 0.001	0.01 ± 0.01	< 0.001	0.43 ± 0.07	< 0.001

Multiple analysis concerning DSQ scales (Table 5) revealed an independent association of duration of disease with “maladaptive action scale”. Greater scores on “self-sacrificing scale” were found for women and patients with multiple lesions. WHO-DAS II and multiple lesions were the only independent factors for the “adaptive scale”. Specifically, patients with multiple lesions scored higher on the “adaptive scale”, while WHO-DAS II was negatively associated with the “adaptive scale”.

**Table 5:** Multiple Linear Regression Models for DSQ Scales: Regression Coefficients ± Standard Error

	Maladaptive action		Image distorting		Self-sacrificing		Adaptive	
	β ± SE	P	β ± SE	P	β ± SE	P	β ± SE	P
Sex								
Men, reference								
Women	13.8 ± 12.9	0.291	12.47 ± 7.47	0.102	8.26 ± 3.31	0.017	2.04 ± 2.96	0.495
Age (years)	0.75 ± 0.59	0.213	0.35 ± 0.34	0.310	0.16 ± 0.15	0.285	-0.01 ± 0.14	0.970
Duration of disease	1.16 ± 0.52	0.032	0.15 ± 0.32	0.653	0.25 ± 0.14	0.090	0.20 ± 0.12	0.095
Multiple lesions	9.95 ± 10.66	0.356	-2.81 ± 6.17	0.651	6.29 ± 2.38	0.012	6.07 ± 2.21	0.009
ASA								
I-II, reference								
III-IV	20.39 ± 11.36	0.080	11.69 ± 6.57	0.082	3.89 ± 2.91	0.189	-1.51 ± 2.61	0.565
WHODASII	-0.05 ± 0.28	0.861	-0.18 ± 0.16	0.274	-0.01 ± 0.06	0.365	-0.15 ± 0.06	0.022

## Discussion

According to the natural history of the ONFH, early symptoms of the disease are characterized by an insidious onset of poorly localized and vague ache around the hip joint, at the lower pelvis, at the medial aspect of thigh and at the buttocks. Later symptoms become more severe and include pain with movement of the hip, difficulties in physical function and limping and restriction of hip range of motion [3]. Taking into account that ONFH affects young individuals with increased needs of mobility and physical activity, their symptoms lead to a considerable deterioration in their daily life, which could subsequently induce psychological effects. Some researchers support the hypothesis that global disability produced from trauma [20] or other pathology [21] of the musculoskeletal system can induce psychological distress such as depression or anxiety.

The results of the present study revealed that patients with ONFH experience psychological distress with depressive symptoms (feelings and somatic symptoms) and social disability compared to healthy controls.

Symptoms of depression are more common in patients with long duration of disease and in patients having more serious and life-threatening basic/primary disease (ASA III or IV), while patients of older age at the onset of disease and patients with multiple lesions present a higher level of anxiety. It seems that the ONFH is a chronic recalcitrant and debilitating disease with long duration of symptoms making patients vulnerable to psychological effects.

Hadjigeorgiou et al suggested that non-traumatic ONFH is indeed a multisystem microvascular disorder rather than a human skeleton disease [9]. Our results are in agreement with findings from investigation of multisystem diseases like SS or early rheumatoid arthritis (RA). Patients with SS present “depressive feelings”, or demonstrate true depressive symptoms [22-25]. Similarly, patients with early RA, a systemic autoimmune disease, express psychological distress with somatic symptoms of depression, feelings of depression and social dysfunction [26], or experience depressed mood [27, 28]. The above findings are in agreement with our results. However, concerning RA, other researchers argue that there is no significant relationship between psychiatric symptoms and measures of disease activity [29-31].

Patients with ONFH seem to present two types of personality when compared to control group. This appears to be the case considering the prevalence of the defence styles of image-distorting and self-sacrificing in the individuals of our sample. The self-sacrificing action style is predominant especially among women and patients having multiple lesions. These organizations of personality are not in agreement with the results of other researchers, who suggest that the maladaptive profile is rather associated with depression in somatic illnesses [16, 24]. Only the duration of the ONFH is associated with the maladaptive action style of the personality in our sample, which means that patients who have inability to deal with their impulses by taking constructive action, on their own behalf, experience a longer duration of their disease.

At last, the functional disability of the patients with ONFH of our sample is positively associated with all the dimensions of psychological distress as measured by GHQ scale and negatively with the adaptive organization of personality. The above finding poses a problem: the psychological distress is a consequence of the functional disability or vice versa? On the other hand, functional disability in our sample is associated with a non-structured personality style with adaptive traits.

The relative small sample size and the use of self-reported questionnaire to investigate the psychological distress do not permit us to generalize our findings. Moreover, since common somatic symptoms of depression investigated by GHQ-28 are similar with early symptoms of ONFH lesions in the skeleton, it is difficult to distinguish between somatic symptoms and the more specific symptoms of depression.

The differences observed between patients and healthy controls do not necessarily express differences between patients suffering from ONFH and the general population, since subjects having any medical or psychiatric problem were excluded from the control group.

In conclusion, patients suffering from ONFH perceive limitations in their physical routine and daily activities, together with restrictions in the social participation, probably leading to a psychological distress. Their personality organization becomes an equally important factor with regard to their functional disability. Patients with an adaptive organization of their personality, present with lesser limitations in their daily life activities. Further longitudinal studies are needed to investigate whether personality traits and psychological distress could influence the functional disability and the clinical course of patients suffering from ONFH and probably to determine factors that may protect patients from the negative consequences of their disease.
